# Use of Hyperoncotic Human Albumin Solution in Severe Traumatic Brain Injury Revisited—A Narrative Review and Meta-Analysis

**DOI:** 10.3390/jcm11092662

**Published:** 2022-05-09

**Authors:** Christian J. Wiedermann

**Affiliations:** 1 Institute of General Practice and Public Health, Claudiana—College of Health Professions, 39100 Bolzano, Italy; christian.wiedermann@am-mg.claudiana.bz.it; 2 Department of Public Health, Medical Decision Making and HTA, University of Health Sciences, Medical Informatics and Technology, 6060 Hall in Tyrol, Austria

**Keywords:** albumin, brain injury, intracranial pressure, oncotic pressure, resuscitation

## Abstract

A significant proportion of patients with a severe traumatic brain injury (TBI) have hypoalbuminemia and require fluid resuscitation. Intravenous fluids can have both favorable and unfavorable consequences because of the risk of hyperhydration and hypo- or hyperosmolar conditions, which may affect the outcome of a TBI. Fluid resuscitation with human albumin solution (HAS) corrects low serum albumin levels and aids in preserving euvolemia in non-brain-injured intensive care units and in perioperative patients. However, the use of HAS for TBI remains controversial. In patients with TBI, the infusion of hypooncotic (4%) HAS was associated with adverse outcomes. The side effects of 4% HAS and the safety and efficacy of hyperoncotic (20–25%) HAS used in the Lund concept of TBI treatment need further investigation. A nonsystematic review, including a meta-analysis of controlled clinical trials, was performed to evaluate hyperoncotic HAS in TBI treatment. For the meta-analysis, the MEDLINE and EMBASE Library databases, as well as journal contents and reference lists, were searched for pertinent articles up to March 2021. Four controlled clinical studies involving 320 patients were included. The first was a randomized trial. Among 165 patients treated with hyperoncotic HAS, according to the Lund concept, 24 (14.5%) died vs. 59 out of 155 control patients (38.1%). A Lund concept intervention using hyperoncotic HAS was associated with a significantly reduced mortality (*p* = 0.002). Evidence of the beneficial effects of fluid management with hyperoncotic HAS on mortality in patients with TBI is at a high risk of bias. Prospective randomized controlled trials are required, which could lead to changes in clinical practice recommendations for fluid management in patients with TBI.

## 1. Introduction

Despite continuous advances in intensive care, the mortality and permanent disability rates after head injuries remain high [1,2]. Ischemia, hypotension, hypoxia, and energy dysfunction are important determinants of the outcomes following a severe traumatic brain injury (TBI). Cerebral edema and increased intracranial pressure (ICP) are frequently observed after TBI, leading to cerebral ischemia. A complex series of pathological events triggers the propagation of this secondary injury cascade to cerebral areas that are initially not involved in TBI [3]. The mortality among patients with TBI is significantly increased in the presence of intracranial hypertension (ICP ≥ 20 mmHg), regardless of the cerebral perfusion pressure (CPP) [4], and patient management has focused on preventing or ameliorating the secondary injury that occurs in the ensuing hours and days following the primary initial trauma. An overarching goal of medical management is to ensure optimal cerebral perfusion and oxygenation [5].

Intravenous fluids play a central role in the management of TBI, allowing adequate CPP to be maintained and helping to avoid intracerebral edema and elevated ICP. However, fluids can have both favorable and unfavorable consequences because of the potential risks of hyperhydration and hypo- or hyperosmolar conditions, which may affect the clinical course and outcome of TBI [6]. 

### Use of Human Albumin Solutions in Fluid Management of TBI

Recent evidence of the low risk of bias confirmed that the use of 20–25% (hyperoncotic) human albumin solution (HAS) to correct low serum albumin levels aids in preserving euvolemia in non-brain-injured intensive care units and perioperative patients [7,8]. Previously, an infusion of 25% HAS was shown to prevent intracerebral edema in patients with TBI [9] and decrease ICP after craniotomy [10].

An ICP-targeted treatment concept for TBI was developed by investigators in Lund, Sweden, utilizing hyperoncotic HAS to maintain euvolemia and colloid osmotic pressure [11]. However, HAS as a replacement fluid in acute brain injury patients is not used in most centers worldwide and is not recommended in international clinical guidelines because of reports of adverse outcomes of HAS infusion [12,13,14]. Specifically, the SAFE-TBI study (a post-hoc follow-up analysis of 290 patients from the randomized SAFE trial) reported higher mortality in those receiving 4% HAS [15]. The authors of this post-hoc analysis suggested that increased albumin may have crossed the damaged blood–brain barrier into the brain tissue, resulting in a greater net outflow of fluid from the cerebral intravascular space into the interstitial brain tissue. An increase in cerebral edema, increase in cerebral pressure, more frequent use of cerebral pressure-lowering measures and, finally, increased mortality in the 4% HAS group compared to the group receiving physiological saline for volume therapy were observed [15,16]. 

These findings remain the subject of debate, because the patients were not enrolled in the SAFE study according to any specific set of TBI-related criteria, and the use of a hypotonic preparation of 4% HAS, particularly in conjunction with the liberal use of vasopressors and relatively high hydrostatic pressure, may have been suboptimal for the patients with severe TBI [17]. The mean change in ICP from randomization to 14 days post-randomization was subsequently analyzed in a post-hoc subgroup of 209 patients of the 290 patient SAFE-TBI study subgroup associated with the use of 4% HAS with increased ICP on day 7 but not on day 3, day 14, or overall [18]. In this subgroup analysis of a subgroup, the initial mean ICP was 21% higher in the group allocated to 4% HAS (*p* = 0.06), and no attempt was made to adjust for this imbalance [18]. Experimental findings directly comparing the commercially available hypotonic 4% HAS used in the SAFE study (4% Albumex (278 mOsm/kg)) with a novel isotonic 4% HAS (288 mOsm/kg) finally confirmed that the tonicity of 4% HAS, rather than the albumin itself, was responsible for increasing the ICP [19].

A recently published BaSICS study in Brazil confirmed this hypothesis [20]. When comparing a balanced infusion solution (Plasma-Lyte 148^®^, Baxter Hospitalar, Brazil) to isotonic saline, a subgroup analysis of the patients with TBI showed that a significantly higher 90-day survival rate was observed under isotonic saline than in patients treated with the balanced solution [21]. Compared to the 0.9% saline solution, the balanced solution used had a theoretical osmolarity of 296 vs. 308 mOsmol/L, whereas the measured osmolality showed an osmolar difference of 271 vs. 296 mOsmol/kgH_2_O [22]. 

The European Society of Intensive Care Medicine (ESICM) consensus and clinical practice recommendations suggest against the use of 4% or 20% HAS as the resuscitation fluid in acute brain injury patients with low blood pressure independent of HAS tonicity (weak recommendation) [14], despite the existence of suggestive evidence that the Lund concept of normalization of plasma oncotic pressure with slowly infused 20–25% HAS may lower the mortality rate compared with alternative approaches in TBI. Several studies have consistently reported low mortality rates ranging from 8% to 20% in patients with severe TBI [23,24,25,26,27,28,29,30,31,32,33], whereas the mean percentages of all injury-related mortality caused by or associated with TBI in Europe and the United States are 37% and 30.5%, respectively [34].

As the Lund concept offers to further characterize the clinical usefulness of 20–25% HAS in patients after TBI, this clinical review focuses on controlled studies directly comparing the Lund concept with an alternative treatment strategy. The studies were relatively few in number and small in size. Hence, there is considerable uncertainty regarding the comparative impact of the Lund concept on mortality. Therefore, it is of substantial interest to consider studies designed to evaluate fluid regimens that incorporate a HAS infusion in patients with severe TBI. The present study quantitatively combines mortality data from such studies in a nonsystematic review.

## 2. Methods

The review article was reported in accordance with the scale for the assessment of narrative review articles (SANRA) [35]. Controlled clinical studies, both randomized and nonrandomized, were eligible for quantitative analysis if they compared the Lund concept with an alternative treatment strategy for patients with severe TBI. Data on mortality and long-term neurological outcomes were available. No limitations were placed on the language of reporting or the time period during which the study was conducted. Non-English candidate studies were translated as required. Randomized and nonrandomized studies were included. When feasible, the inclusion of nonrandomized studies in meta-analyses has been recommended, because they can increase the statistical power and permit important clinical questions to be addressed for which randomized trial data are unavailable or inadequate [36,37]. Published studies were searched using multiple methods without language and time restrictions, including computer searches of MEDLINE and EMBASE. The reference lists of online journals were also examined. A representative MEDLINE search strategy is shown in Table 1. 

The determination of the study eligibility and data extraction for the statistical analyses were performed in a nonsystematic manner. The endpoint was the relative risk (RR) for mortality. Heterogeneity was assessed using the Cochran Q test and I^2^ statistics [38]. The results were quantitatively combined using a random effects model [39]. RR for individual studies and pooled RR were computed with 95% confidence intervals (CI). Linear regression of the standardized effect vs. precision was used to evaluate the possible publication bias [40]. Analyses were performed using Comprehensive Meta-Analysis Version 2.2.64 (Biostat Inc., Englewood, NJ, USA).

## 3. Results

### 3.1. Clinical Trials on Fluid Regimens Incorporating Hyperoncotic HAS Infusion in Severe TBI

A decrease in intracranial pressure from 25% HAS infusion has been previously reported [10]. The administration of 25% HAS prevented or reduced cerebral edema in a nonrandomized controlled study [41] and two randomized trials using 20% and 25% HAS, respectively [9,42]. Twelve-month mortality rates below 20% have been attained in single-arm cohort studies using the Lund concept in patients with severe TBI who received hyperoncotic HAS for their fluid replacement [25,31].

For a meta-analysis of controlled clinical studies of ICP-targeted treatment for severe TBI in the ICU, including the administration of concentrated HAS after the Lund concept, 398 candidate reports were identified in MEDLINE on 30 March 2021. In addition, 55 additional reports were identified in EMBASE using a corresponding search strategy and/or a manual search of the reference lists. Of these 453 reports, 84 were found to satisfy the eligibility criteria upon screening and were retrieved and examined in detail. At that stage, 76 reports were excluded from the quantitative analysis, most often because they consisted of literature reviews with no original data or involved studies that did not evaluate the Lund concept or lacked a control group. Data from one study [24] appeared in four additional reports [23,43,44,45], leaving four studies for the meta-analysis. The risk of bias of the studies was assessed using the NIH National Heart, Lung, and Blood Institute Quality Assessment of Controlled Intervention Studies [46] tool.

**Figure 1:**
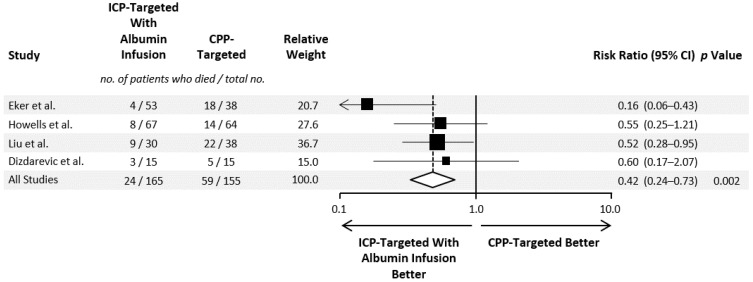
Meta-analysis of mortality in controlled clinical studies from Eker et al [23,24,43,44,45], Howells et al. [49], Liu et al. [47], and Dizdarevic et al. [48] comparing the intracranial pressure-targeted Lund concept treatment, including albumin infusion, with cerebral perfusion pressure-targeted therapy of severe traumatic brain injury in adults. A random effects model was used for the analysis. The size of the squares indicates the data points from the individual studies scaled according to the percentage of the total weight (with individual trial weights equaling the proportion of total patients receiving Lund concept treatment multiplied by the number of deaths in the cerebral perfusion pressure-targeted group), and the diamond indicates the pooled findings. The dashed line indicates pooled relative risk. The proportion of the variation attributable to heterogeneity (I2) was 38.6% (95% CI, 0.0–79.0%). CPP, cerebral perfusion pressure; CI, confidence interval.

In four controlled clinical studies of severe TBI, the Lund concept was compared with cerebral perfusion pressure (CPP)-targeted therapy (Table 2 and Table 3) [24,47,48,49]. One study was a randomized controlled trial of 60 patients with acute brain injury, including 30 patients with TBI [48]. One nonrandomized study compared patients treated according to the Lund concept at one center with those receiving CPP-targeted therapy at another center [49]. Two nonrandomized studies compared the outcomes of patients treated at the same hospital before and after switching from CPP-targeted therapy to the Lund concept [24,47]. In all four studies, mortality was lower in the patients receiving 20–25% HAS as part of the Lund concept treatment, and their respective RR ranged from 0.16 to 0.60 (Figure 1). Of the 165 patients treated using the Lund concept, 24 (14.5%) died, compared with 59 out of 155 patients (38.1%) receiving CPP-targeted therapy. No significant heterogeneity was observed in the RR for mortality between the studies (*p* = 0.18), and no evidence of publication bias was found (*p* = 0.65). The pooled RR for mortality was 0.42 (95% confidence interval, 0.24–0.73; *p* = 0.002), indicating a 58% RR reduction associated with the adoption of the Lund concept. All studies used in the meta-analysis had a high risk of bias (Table 4).

### 3.2. Evidence Synthesis of Hyperoncotic HAS Administration in TBI

The present nonsystematic review suggests that therapy for severe TBI with 20–25% HAS in the context of the Lund concept can improve mortality. Lower mortality in the Lund concept groups of all four included studies and similar mortality rates in the Lund and CPP-targeted groups in the meta-analysis, as in previously reported single-arm cohorts treated according to these two strategies despite widely geographically dispersed settings and different time periods, suggest internal and external consistencies, respectively. However, the relatively small number and size of available controlled studies is a major limitation of this analysis. 

Only four studies were identified for inclusion in this report, and only one of them was a randomized trial. This trial [48] was the only randomized evaluation of the Lund concept identified in a previous Cochrane review [51]. The Cochrane investigators excluded the trial, because it included not only severe TBI patient data but also subarachnoid hemorrhage patients. The use of historical controls in one study [24] may be another limitation. Moreover, there were inconsistencies in the treatments followed by the Lund concept across the studies included in this meta-analysis. Eker et al. [24] used dihydroergotamine to decrease the intracranial venous blood volume, and Liu et al. [47] administered 20% mannitol to patients with ICP ≥ 20 mmHg, which is no longer recommended in Lund concept treatment and is followed by a rebound increase in ICP. A modified Lund concept was used by Dizdarevic et al. [48]. Despite these treatment inconsistencies, all the studies included in this meta-analysis used hyperoncotic HAS for plasma volume expansion.

These studies did not meet the criteria for a robust design and reporting. Although the inclusion of both randomized and nonrandomized studies in a meta-analyses is recommended [36,37], nonrandomized studies can be vulnerable to biases. Nevertheless, the Lund and control groups in the nonrandomized studies of this meta-analysis were well-matched for the baseline risk factors of sex, age, and Glasgow Coma Scale scores (Table 3). Since there is a lack of new studies on the treatment of TBI with HAS, many of the cited references were old, which was an additional limitation of this review. Hence, the currently available evidence from controlled clinical studies suggests that therapy for severe TBI under the Lund concept can improve the outcomes; however, the evidence is limited in scope and quality.

## 4. Mechanistic Considerations for the Use of Hyperoncotic HAS in TBI

The physiological considerations of intravenous HAS as a replacement fluid and the extant clinical evidence for and against its use within the various facets of modern neuroanesthesia and neurocritical care practice were recently explored and reviewed by Ma and Bebawy [13]. The recommendation was made so that, in the absence of definitive data to either support or dissuade from the use of HAS in most neurosurgical scenarios, practitioners should consider the potential risks and benefits of HAS administration. In the narrative review, no mention was made of the ICP-targeted treatment of TBI utilizing 20–25% HAS [13], suggesting that data on HAS administration in the context of the Lund concept have not been taken into consideration.

HAS infusion to maintain normal serum albumin levels is the cornerstone of the Lund concept [11,12]. HAS is an effective volume expander and, along with erythrocyte transfusions, aids in preserving euvolemia, reducing reliance on vasopressors, and thereby averting intracranial hypertension. Additionally, as the chief endogenous colloid of human plasma, albumin sustains oncotic forces that retain the fluid in the intravascular compartment, consequently minimizing tissue edema in the injured brain and the rest of the body. The administration of concentrated albumin prevented or reduced cerebral edema in two randomized trials [9,42] and in a nonrandomized controlled study [41].

Research has identified a wide range of putative roles for HAS in modifying inflammation, maintaining vascular endothelial integrity and the acid–base balance, and ligating endogenous and exogenous compounds [52], which may all play important roles in the pathophysiology of severe TBI. Albumin can offer protection from inflammatory processes and the associated damage to the microcirculation and tissues, with an impact on the outcome [53].

In addition, supporting the utility of HAS is the observation that hypoalbuminemia is independently associated with increased mortality among severe TBI patients [54]. The kinetics of albumin involves a transcapillary leak and breakdown, leading to hypoalbuminemia, which is associated with the worse outcomes in a broad spectrum of conditions [55]. The correction of hypoalbuminemia with hyperoncotic HAS infusion can be beneficial, as it improves the hemodynamic stability in patients with sepsis [56] and prevents acute kidney injury in cardiac surgery patients [57]. Intravenous hyperoncotic HAS has been determined to be safe for use as resuscitation fluid in most critically ill patients [7].

Neuroinflammation is recognized as an interaction between central and peripheral components that is influenced by age, sex, type of TBI and its severity, and other factors, including the timing of the diagnostic and therapeutic interventions that may have a significant impact on the outcome [58]. Although HAS therapy in TBI may have neuroprotective potential [59], no data supporting this hypothesis are currently available. Moreover, the colloids used in the Lund concept were not restricted to hyperoncotic 20% HAS but also included 4% HAS, plasma, and packed red blood cells (no synthetic colloids were used) [60]. If the timing of 20% HAS administration, i.e., early vs. late in TBI, is important remains speculative.

## 5. Conclusions

The use of HAS for TBI is not recommended in most fluid management guidelines and remains controversial. Evidence of the beneficial effects of fluid management with HAS solution on relevant clinical outcomes in patients with severe TBI is largely observational. There have been no studies with a low risk of bias performed to evaluate the Lund guidelines relative to any alternative guidelines, as there are no studies that support any specific TBI guidelines [12]. There have been several studies with a high risk of bias, providing some support for Lund therapy. The use of 20–25% HAS according to the Lund concept was associated with significantly reduced mortality in four small controlled clinical trials that were heterogeneous in their design and all at a high risk of bias. In the SAFE-TBI study, a higher mortality in TBI patients receiving 4% HAS was observed; however, the evidence suggested that the tonicity of HAS, rather than albumin itself, was responsible for adverse the outcomes by increasing the ICP. Further clinical studies are warranted to define the benefits of 20–25% HAS in TBI, as in the Lund concept of fluid replacement for euvolemia with the normalization of plasma oncotic pressure using hyperoncotic HAS, which, according to this meta-analysis, does not increase mortality. Prospective randomized controlled trials are required and, if these hypotheses are confirmed, could lead to changes in clinical practice recommendations for fluid management in patients with TBI. 

## Figures and Tables

**Table 1:** Representative the MEDLINE search strategy.

Set	Query
1	brain OR head OR cerebr* OR cranial OR intracranial
2	injur* OR trauma* OR contusion* OR concussion* OR damage OR herniat*
3	#1 AND #2
4	Lund [tiab] OR “intracranial pressure-targeted” OR “ICP-targeted”
5	mortality OR surviv* OR death* OR died OR neurological outcome OR “Glasgow outcome scale” OR GOS
6	random* [tiab] OR “random allocation” [mh] OR “randomized controlled trial” [pt]
7	control* [tiab] OR “controlled clinical trial” [pt]
8	#6 OR #7
9	#3 AND #4 AND #5 AND #8

**Table 2:** Attributes of the studies included in the meta-analysis.

Study	Patients	Indication	Fluid Regimen
Eker et al. 1998 [23,24,43,44,45]	91	Head injury with GCS < 8 and ICP > 25 mm Hg	ICP-targeted therapy with albumin infusion to maintain serum albumin ≤ 40 g·L^−1^ vs. conventional treatment
Howells et al. 2005 [49]	131	Head injury requiring at least 6 h of ICP, CPP, and MAP data recorded within 96 h of injury	ICP-targeted therapy with albumin infusion to maintain adequate COP, stable MAP, and CVP ≤ 5 mmHg * vs. CPP-targeted therapy
Liu et al. 2010 [47]	68	Head injury and mean GCS of 5.8	ICP-targeted therapy with albumin infusion to maintain serum albumin ≤ 40 g·L^−1^ vs. CPP-targeted therapy
Dizdarevic et al. 2012 [48]	30	Isolated head injury and intradural focal lesions with GCS ≤ 8 and secondary brain ischemia	ICP-targeted therapy with albumin infusion to maintain a serum albumin of approximately 40 g·L^−1^ vs. CPP-targeted therapy

* Albumin infusion specified in Elf et al. [50]. Abbreviations: MAP, mean arterial pressure; COP, colloid osmotic pressure; CPP, cerebral perfusion pressure; CVP, central venous pressure; GCS, Glasgow Coma Scale; ICP, intracranial pressure.

**Table 3:** Baseline data of the patients in the meta-analysis.

Study	Males, *n* (%)		Age (y) *		GCS *	
	ICP-Targeted with Albumin Infusion	CPP-Targeted	ICP-Targeted with Albumin Infusion	CPP-Targeted	ICP-Targeted with Albumin Infusion	CPP-Targeted
Eker et al. 1998 [23,24,43,44,45]	n.d.	30 (78.9)	Grouped ^†^	20 (7–59) ^‡^	<8	coma > 6 h
Howells et al. 2005 [49]	n.d.	n.d.	40 ± 18	39 ± 18	4.5 ± 1.1^††^	3.5 ± 1.6 ^††^
Liu et al. 2010 [47]	17 (56.7)	28 (73.7)	53.3 ± 20.3	55.6 ± 19.8	5.9 ± 1.4	5.7 ± 1.3
Dizdarevic et al. 2012 [48]	10 (66.7)	12 (80.0)	35.7 ± 17.7	43.0 ± 14.8	5 ^§^	5 ^§^

* Mean ± SD unless otherwise indicated. ^†^ Twenty-three patients (43.4%) < 21 y of age, 20 (37.7%) 21–40 y, 9 (17.0%) 41–60 y, and 1 (1.9%) > 60 y. ^‡^ Median (range). ^§^ Mean only, SD not reported. ^††^ GCS motor scale. Abbreviations: CPP, cerebral perfusion pressure; GCS, Glasgow Coma Scale; SD, standard deviation; n.d., no data.

**Table 4:** Risk of bias assessment of the studies in the meta-analysis using the NIH National Heart, Lung, and Blood Institute Quality Assessment of Controlled Intervention Studies [46] tool.

Criteria		Eker et al. 1998 [23,24,43,44,45]	Howells et al. 2005 [49]	Liu et al. 2010 [47]	Dizdarevic et al. 2012 [48]
1.	Was the study described as randomized, a randomized trial, a randomized clinical trial, or an RCT?	No	No	No	Yes
2.	Was the method of randomization adequate (i.e., use of randomly generated assignment)?	NA	NA	NA	Yes
3.	Was the treatment allocation concealed (so that assignments could not be predicted)?	NA	NA	NA	Yes
4.	Were study participants and providers blinded to treatment group assignment?	No	No	No	No
5.	Were the people assessing the outcomes blinded to the participants’ group assignments?	No	No	No	No
6.	Were the groups similar at baseline on important characteristics that could affect outcomes (e.g., demographics, risk factors, co-morbid conditions)?	Yes	Yes	Yes	Yes
7.	Was the overall drop-out rate from the study at endpoint 20% or lower of the number allocated to treatment?	NR	NR	NR	NR
8.	Was the differential drop-out rate (between treatment groups) at endpoint 15 percentage points or lower?	NR	NR	NR	NR
9.	Was there high adherence to the intervention protocols for each treatment group?	NR	NR	NR	NR
10.	Were other interventions avoided or similar in the groups (e.g., similar background treatments)?	No	No	No	No
11.	Were outcomes assessed using valid and reliable measures, implemented consistently across all study participants?	Yes	Yes	Yes	Yes
12.	Did the authors report that the sample size was sufficiently large to be able to detect a difference in the main outcome between groups with at least 80% power?	No	No	No	No
13.	Were outcomes reported or subgroups analyzed prespecified (i.e., identified before analyses were conducted)?	Yes	Yes	Yes	Yes
14.	Were all randomized participants analyzed in the group to which they were originally assigned, i.e., did they use an intention-to-treat analysis?	NA	NA	NA	No
Risk of bias		High	High	High	High

Abbreviations: NA, not applicable; NR, not reported.

## Data Availability

Data for this narrative review and meta-analysis are available from the author upon request.

## References

[1] Patel H.C., Bouamra O., Woodford M., King A.T., Yates D.W., Lecky F.E. (2005). Trends in Head Injury Outcome from 1989 to 2003 and the Effect of Neurosurgical Care: An Observational Study. Lancet.

[2] Beck B., Gantner D., Cameron P.A., Braaf S., Saxena M., Cooper D.J., Gabbe B.J. (2018). Temporal Trends in Functional Outcomes after Severe Traumatic Brain Injury: 2006–2015. J. Neurotrauma.

[3] Kinoshita K. (2016). Traumatic Brain Injury: Pathophysiology for Neurocritical Care. J. Intensive Care.

[4] Juul N., Morris G.F., Marshall S.B., Marshall L.F. (2000). Intracranial Hypertension and Cerebral Perfusion Pressure: Influence on Neurological Deterioration and Outcome in Severe Head Injury. The Executive Committee of the International Selfotel Trial. J. Neurosurg..

[5] Stocchetti N., Carbonara M., Citerio G., Ercole A., Skrifvars M.B., Smielewski P., Zoerle T., Menon D.K. (2017). Severe Traumatic Brain Injury: Targeted Management in the Intensive Care Unit. Lancet Neurol..

[6] Rossi S., Picetti E., Zoerle T., Carbonara M., Zanier E.R., Stocchetti N. (2018). Fluid Management in Acute Brain Injury. Curr. Neurol. Neurosci. Rep..

[7] Wiedermann C.J. (2020). Phases of Fluid Management and the Roles of Human Albumin Solution in Perioperative and Critically Ill Patients. Curr. Med. Res. Opin..

[8] Haynes G. (2020). Growing Evidence for Hyperoncotic 20% Albumin Solution for Volume Resuscitation. J. Cardiothorac. Vasc. Anesth..

[9] Tomita H., Ito U., Tone O., Masaoka H., Tominaga B. (1994). High Colloid Oncotic Therapy for Contusional Brain Edema. Acta Neurochirurgica Suppl..

[10] Miyasaka Y., Nakayama K., Matsumori K., Beppu T., Tanabe T., Kitahara T., Saito T. (1983). Albumin therapy for patients with increased intracranial pressure: Oncotic therapy. No Shinkei Geka.

[11] Grände P.-O. (2006). The “Lund Concept” for the Treatment of Severe Head Trauma—Physiological Principles and Clinical Application. Intensive Care Med..

[12] Grände P.-O., Juul N. (2020). Guidelines for Treatment of Patients with Severe Traumatic Brain Injury. Management of Severe Traumatic Brain Injury.

[13] Ma H.K., Bebawy J.F. (2021). Albumin Use in Brain-Injured and Neurosurgical Patients: Concepts, Indications, and Controversies. J. Neurosurg. Anesthesiol..

[14] Oddo M., Poole D., Helbok R., Meyfroidt G., Stocchetti N., Bouzat P., Cecconi M., Geeraerts T., Martin-Loeches I., Quintard H. (2018). Fluid Therapy in Neurointensive Care Patients: ESICM Consensus and Clinical Practice Recommendations. Intensive Care Med..

[15] Myburgh J., Cooper D.J., Finfer S., Bellomo R., Norton R., Bishop N., SAFE Study Investigators, Australian and New Zealand Intensive Care Society Clinical Trials Group, Australian Red Cross Blood Service, George Institute for International Health (2007). Saline or Albumin for Fluid Resuscitation in Patients with Traumatic Brain Injury. N. Engl. J. Med..

[16] Gantner D., Moore E.M., Cooper D.J. (2014). Intravenous Fluids in Traumatic Brain Injury: What’s the Solution?. Curr. Opin. Crit. Care.

[17] Drummond J.C., Patel P.M., Lemkuil B. (2011). Proscribing the Use of Albumin in the Head-Injured Patient Is Not Warranted. Anesth. Analg..

[18] Cooper D.J., Myburgh J., Heritier S., Finfer S., Bellomo R., Billot L., Murray L., Vallance S., SAFE-TBI Investigators, Australian and New Zealand Intensive Care Society Clinical Trials Group (2013). Albumin Resuscitation for Traumatic Brain Injury: Is Intracranial Hypertension the Cause of Increased Mortality?. J. Neurotrauma.

[19] Iguchi N., Kosaka J., Bertolini J., May C.N., Lankadeva Y.R., Bellomo R. (2018). Differential Effects of Isotonic and Hypotonic 4% Albumin Solution on Intracranial Pressure and Renal Perfusion and Function. Crit. Care Resusc. J. Australas. Acad. Crit. Care Med..

[20] Briegel J. (2022). Albumin in traumatic brain injury-osmolarity is what matters!. Med. Klin. Intensivmed. und Notf..

[21] Zampieri F.G., Machado F.R., Biondi R.S., Freitas F.G.R., Veiga V.C., Figueiredo R.C., Lovato W.J., Amêndola C.P., Serpa-Neto A., Paranhos J.L.R. (2021). Effect of Intravenous Fluid Treatment With a Balanced Solution vs 0.9% Saline Solution on Mortality in Critically Ill Patients: The BaSICS Randomized Clinical Trial. JAMA.

[22] Weinberg L., Collins N., Van Mourik K., Tan C., Bellomo R. (2016). Plasma-Lyte 148: A Clinical Review. World J. Crit. Care Med..

[23] Asgeirsson B., Grände P.O., Nordström C.H. (1994). A New Therapy of Post-Trauma Brain Oedema Based on Haemodynamic Principles for Brain Volume Regulation. Intensive Care Med..

[24] Eker C., Asgeirsson B., Grände P.O., Schalén W., Nordström C.H. (1998). Improved Outcome after Severe Head Injury with a New Therapy Based on Principles for Brain Volume Regulation and Preserved Microcirculation. Crit. Care Med..

[25] Naredi S., Edén E., Zäll S., Stephensen H., Rydenhag B. (1998). A Standardized Neurosurgical Neurointensive Therapy Directed toward Vasogenic Edema after Severe Traumatic Brain Injury: Clinical Results. Intensive Care Med..

[26] Naredi S., Olivecrona M., Lindgren C., Ostlund A.L., Grände P.O., Koskinen L.O. (2001). An Outcome Study of Severe Traumatic Head Injury Using the “Lund Therapy” with Low-Dose Prostacyclin. Acta Anaesthesiol. Scand..

[27] Wahlström M.R., Olivecrona M., Koskinen L.-O.D., Rydenhag B., Naredi S. (2005). Severe Traumatic Brain Injury in Pediatric Patients: Treatment and Outcome Using an Intracranial Pressure Targeted Therapy—The Lund Concept. Intensive Care Med..

[28] Olivecrona M., Rodling-Wahlström M., Naredi S., Koskinen L.-O.D. (2007). Effective ICP Reduction by Decompressive Craniectomy in Patients with Severe Traumatic Brain Injury Treated by an ICP-Targeted Therapy. J. Neurotrauma.

[29] Olivecrona M., Rodling-Wahlström M., Naredi S., Koskinen L.-O.D. (2009). Prostacyclin Treatment in Severe Traumatic Brain Injury: A Microdialysis and Outcome Study. J. Neurotrauma.

[30] Olivecrona M., Rodling-Wahlström M., Naredi S., Koskinen L.-O.D. (2012). Prostacyclin Treatment and Clinical Outcome in Severe Traumatic Brain Injury Patients Managed with an ICP-Targeted Therapy: A Prospective Study. Brain Inj..

[31] Gautschi O.P., Huser M.C., Smoll N.R., Maedler S., Bednarz S., von Hessling A., Lussmann R., Hildebrandt G., Seule M.A. (2013). Long-Term Neurological and Neuropsychological Outcome in Patients with Severe Traumatic Brain Injury. Clin. Neurol. Neurosurg..

[32] Stenberg M., Koskinen L.-O., Levi R., Stålnacke B.-M. (2013). Severe Traumatic Brain Injuries in Northern Sweden: A Prospective 2-Year Study. J. Rehabil. Med..

[33] Koskinen L.-O.D., Olivecrona M., Grände P.O. (2014). Severe Traumatic Brain Injury Management and Clinical Outcome Using the Lund Concept. Neuroscience.

[34] Maas A.I.R., Menon D.K., Adelson P.D., Andelic N., Bell M.J., Belli A., Bragge P., Brazinova A., Büki A., Chesnut R.M. (2017). Traumatic Brain Injury: Integrated Approaches to Improve Prevention, Clinical Care, and Research. Lancet Neurol..

[35] Baethge C., Goldbeck-Wood S., Mertens S. (2019). SANRA—a Scale for the Quality Assessment of Narrative Review Articles. Res. Integr. Peer Rev..

[36] Shrier I., Boivin J.-F., Steele R.J., Platt R.W., Furlan A., Kakuma R., Brophy J., Rossignol M. (2007). Should Meta-Analyses of Interventions Include Observational Studies in Addition to Randomized Controlled Trials? A Critical Examination of Underlying Principles. Am. J. Epidemiol..

[37] Stroup D.F., Berlin J.A., Morton S.C., Olkin I., Williamson G.D., Rennie D., Moher D., Becker B.J., Sipe T.A., Thacker S.B. (2000). Meta-Analysis of Observational Studies in Epidemiology: A Proposal for Reporting. Meta-Analysis Of Observational Studies in Epidemiology (MOOSE) Group. JAMA.

[38] Higgins J.P., Thompson S.G. (2002). Quantifying Heterogeneity in a Meta-analysis. Stat. Med..

[39] DerSimonian R., Laird N. (1986). Meta-Analysis in Clinical Trials. Control. Clin. Trials.

[40] Egger M., Davey Smith G., Schneider M., Minder C. (1997). Bias in Meta-Analysis Detected by a Simple, Graphical Test. BMJ.

[41] Tone O., Ito U., Tomita H., Masaoka H., Tominaga B. (1994). High Colloid Oncotic Therapy for Brain Edema with Cerebral Hemorrhage. Acta Neurochir. Suppl..

[42] Gürkan F., Haspolat K., Yaramiş A., Ece A. (2001). Beneficial Effect of Human Albumin on Neonatal Cerebral Edema. Am. J. Ther..

[43] Schalén W., Sonesson B., Messeter K., Nordström G., Nordström C.H. (1992). Clinical Outcome and Cognitive Impairment in Patients with Severe Head Injuries Treated with Barbiturate Coma. Acta Neurochir..

[44] Schalén W., Messeter K., Nordström C.H. (1992). Complications and Side Effects during Thiopentone Therapy in Patients with Severe Head Injuries. Acta Anaesthesiol. Scand..

[45] Eker C., Schalén W., Asgeirsson B., Grände P.O., Ranstam J., Nordström C.H. (2000). Reduced Mortality after Severe Head Injury Will Increase the Demands for Rehabilitation Services. Brain Inj..

[46] Study Quality Assessment Tools|NHLBI, NIH. https://www.nhlbi.nih.gov/health-topics/study-quality-assessment-tools.

[47] Liu C.-W., Zheng Y.-K., Lu J., Yu W.-H., Wang B., Hu W., Zhu K.-Y., Zhu Y., Hu W.-H., Wang J.-R. (2010). Application of Lund concept in treating brain edema after severe head injury. Chin. Crit. Care Med..

[48] Dizdarevic K., Hamdan A., Omerhodzic I., Kominlija-Smajic E. (2012). Modified Lund Concept versus Cerebral Perfusion Pressure-Targeted Therapy: A Randomised Controlled Study in Patients with Secondary Brain Ischaemia. Clin. Neurol. Neurosurg..

[49] Howells T., Elf K., Jones P.A., Ronne-Engström E., Piper I., Nilsson P., Andrews P., Enblad P. (2005). Pressure Reactivity as a Guide in the Treatment of Cerebral Perfusion Pressure in Patients with Brain Trauma. J. Neurosurg..

[50] Elf K., Nilsson P., Enblad P. (2002). Outcome after Traumatic Brain Injury Improved by an Organized Secondary Insult Program and Standardized Neurointensive Care. Crit. Care Med..

[51] Muzevic D., Splavski B. (2013). The Lund Concept for Severe Traumatic Brain Injury. Cochrane Database Syst. Rev..

[52] Ferrer R., Mateu X., Maseda E., Yebenes J.C., Aldecoa C., De Haro C., Ruiz-Rodriguez J.C., Garnacho-Montero J. (2018). Non-Oncotic Properties of Albumin. A Multidisciplinary Vision about the Implications for Critically Ill Patients. Expert Rev. Clin. Pharmacol..

[53] Hariri G., Joffre J., Deryckere S., Bige N., Dumas G., Baudel J.L., Maury E., Guidet B., Ait-Oufella H. (2018). Albumin Infusion Improves Endothelial Function in Septic Shock Patients: A Pilot Study. Intensive Care Med..

[54] Yang T.-J., Fei M.-M., Ye W., Pan A.-J., Liu B. (2013). Effect of albumin and hemoglobin level on prognosis of patients with uncomplicated severe traumatic brain injury: A retrospective cohort study. Chin. Crit. Care Med..

[55] Soeters P.B., Wolfe R.R., Shenkin A. (2019). Hypoalbuminemia: Pathogenesis and Clinical Significance. J. Parenter. Enter. Nutr..

[56] Caironi P., Tognoni G., Masson S., Fumagalli R., Pesenti A., Romero M., Fanizza C., Caspani L., Faenza S., Grasselli G. (2014). Albumin Replacement in Patients with Severe Sepsis or Septic Shock. N. Engl. J. Med..

[57] Lee E.H., Kim W.J., Kim J.Y., Chin J.H., Choi D.K., Sim J.Y., Choo S.J., Chung C.H., Lee J.W., Choi I.C. (2016). Effect of Exogenous Albumin on the Incidence of Postoperative Acute Kidney Injury in Patients Undergoing Off-Pump Coronary Artery Bypass Surgery with a Preoperative Albumin Level of Less than 4.0 g/dL. Anesthesiology.

[58] Marklund N., Sundstrøm T., Grände P.-O., Luoto T., Rosenlund C., Undén J., Wester K.G. (2020). Pharmacological Neuroprotection. Management of Severe Traumatic Brain Injury: Evidence, Tricks, and Pitfalls.

[59] Khatri R., Afzal M.R., Rodriguez G.J., Maud A., Miran M.S., Qureshi M.A., Cruz-Flores S., Qureshi A.I. (2018). Albumin-Induced Neuroprotection in Focal Cerebral Ischemia in the ALIAS Trial: Does Severity, Mechanism, and Time of Infusion Matter?. Neurocrit. Care.

[60] Rodling Wahlström M., Olivecrona M., Nyström F., Koskinen L.-O.D., Naredi S. (2009). Fluid Therapy and the Use of Albumin in the Treatment of Severe Traumatic Brain Injury. Acta Anaesthesiol. Scand..

